# The fate and function of the *Arabidopsis* Class I formin AtFH1 during central vacuole biogenesis in the rhizodermis

**DOI:** 10.3389/fpls.2025.1685260

**Published:** 2025-10-21

**Authors:** Helena Kočová, Viktor Žárský, Fatima Cvrčková

**Affiliations:** Department of Experimental Plant Biology, Faculty of Science, Charles University, Prague, Czechia

**Keywords:** At3g25500, cytoplasmic streaming, formin, fluorescent protein pH sensitivity, protein degradation, root tip, vacuole configuration, tonoplast

## Abstract

**Introduction:**

During plant cell differentiation, major restructuring of endomembranes takes place, resulting in the formation of the large central vacuole. We previously reported that the *Arabidopsis* Class I formin AtFH1 (At3g25500), a member of a plant-specific family of cytoskeletal organizers that are at the same time integral membrane proteins, transiently localizes to the tonoplast in the root transition zone around the time of central vacuole consolidation.

**Methods:**

We used *in vivo* fluorescent protein expression in combination with pharmacological treatments and quantitative image analysis to examine the behavior and role of AtFH1 during central vacuole formation.

**Results:**

The protein was found to enter the vacuole lumen in the root elongation zone and some shoot tissues, suggesting that its brief residence at the tonoplast in the transition zone is also an intermediate of a process leading to its vacuolar degradation. However, impairment of AtFH1 function by mutations or by the formin inhibitor SMIFH2 resulted in altered vacuole organization, while only the loss-of-function mutations affected rapid, cytoplasmic streaming-related tonoplast motility.

**Discussion:**

These observations suggest that tonoplast-associated AtFH1 may act as an active cargo that affects tonoplast organization while on the way toward being degraded.

## 1 Introduction

The plant vacuole is a compartment with a key role in plant growth and development since it allows rapid, turgor-driven cell expansion without great investments into cytoplasmic components (Dünser et al., 2019). The large central vacuole, present in the majority of differentiated cells of the vegetative plant body, is formed through extensive rearrangements of precursor endomembrane compartments already present in meristematic cells. The *Arabidopsis thaliana* root tip, whose well-characterized developmental zonation allows easy identification of distinct developmental stages, has emerged as an effective model for the study of central vacuole biogenesis (e.g., Oda et al., 2009; Cui et al., 2019; Scheuring et al., 2024). In addition to the structural role involving cell expansion and provision of hydraulic stiffness, the vacuole also plays an important part in other cellular processes, such as protein degradation, storage, or ion homeostasis (Kaiser and Scheuring, 2020; see also Kočová et al., 2025).

There are several pathways whereby a cellular component can enter the vacuole lumen. Small molecules or ions are transported via more or less specific channels or transporters, frequently powered by electrochemical gradient generated by proton pumps—vacuolar ATPase (VHA) and pyrophosphatase (VHP; summarized in Martinoia et al., 2000)—while luminal (vacuolar intrinsic) proteins, as well as the tonoplast resident ones, are mostly delivered by vesicular trafficking through the Golgi apparatus and *trans*-Golgi network (TGN), although alternative pathways bypassing the Golgi apparatus, often termed “unconventional protein secretion”, exist (Pereira et al., 2014; Ivanov and Robinson, 2018).

Vacuoles also serve as a major lytic compartment, i.e., the terminal destination for cellular components that are to be degraded. Cytoplasmic components, including damaged organelles, are engulfed into autophagosomes by macroautophagy (Yoshimoto and Ohsumi, 2018), while membrane-bound proteins need to travel through endosomes, where they are sorted by ESCRT (endosomal sorting complex required for transport) into multivesicular bodies (MVBs; Paez Valencia et al., 2016; Isono and Kalinowska, 2017). Both autophagosomes and MVBs then fuse with the vacuolar membrane and release their cargo for degradation. Alternatively, cargo can be directly engulfed by the tonoplast membrane in a process called microautophagy (Sieńko et al., 2020). Prior to degradation in the vacuole lumen, membrane proteins are freed from the phospholipid bilayer by a mechanism not yet characterized in plants (Ivanov and Robinson, 2018; Yagyu and Yoshimoto, 2024), but possibly analogous to autophagic membrane lysis described in yeast (e.g., Kagohashi et al., 2023).

The tonoplast membrane is surrounded by actin microfilaments, which may indirectly participate in maintaining tonoplast structure, especially in the constricted tubular regions (Kutsuna et al., 2003). Additionally, the inhibition of actin polymerization by Latrunculin B (LatB) results in fragmented, inflated vacuolar compartments (Scheuring et al., 2016). Therefore, proteins or protein complexes mediating microfilament attachment to the tonoplast may modulate vacuole shape. Tonoplast organization is affected not only by the cell’s differentiation status but also by various external or internal conditions, such as nutrition status (Osorio-Navarro et al., 2025), pH, or stiffness of the surrounding media (Dünser et al., 2019). For example, NET4A, a member of the Networked (NET) family of microfilament-binding proteins (Deeks et al., 2012), associates with the tonoplast and contributes to vacuole compactness through interaction with tonoplast Rab7 GTPase RABG3 (Kaiser et al., 2019; Hawkins et al., 2023).

Interesting candidates for possible modulators of vacuole development can be found among transmembrane Class I formins. While the family of formins (FH2 proteins, named after their hallmark actin-nucleating formin homology 2, or FH2, domain) is evolutionarily ancient, only its plant Class I clade typically contains integral membrane proteins that can act as tethers between the actin cytoskeleton and membrane compartments, or even the cell wall in the case of plasmalemma-localized formins (reviewed in Cvrčková et al., 2024). The *Arabidopsis* housekeeping Class I formin AtFH1, as well as its closest relative AtFH2, typically localize to the plasma membrane or intracellular compartments, with enrichment at plasmodesmata (Diao et al., 2018; Oulehlová et al., 2019). In the root tip, whose well-characterized structure allows the identification of distinct developmental zones (Verbelen et al., 2006), the fate of AtFH1 in the course of cell division, expansion, and differentiation can be followed. Remarkably, in the root tip transition zone, GFP (green fluorescent protein)-tagged AtFH1 transiently localizes to the tonoplast around the time of central vacuole development before disappearing completely in the elongation zone (Oulehlová et al., 2019), although its mRNA is detectable in most tissues (Hruz et al., 2008), raising the question of whether the tonoplast-localized AtFH1 participates in central vacuole biogenesis or whether it merely passes through the tonoplast on the way toward degradation.

In this report, we employed additional fluorescent protein markers and pharmacological treatments to confirm that AtFH1 undergoes vacuolar degradation. However, at the same time, we also documented that mutational disruption of AtFH1, as well as the pharmacological inhibition of formin function, alters vacuole organization. Thus, we showed that the Class I formin AtFH1 fits the definition of an active cargo of membrane trafficking in the sense of our recently formulated hypothesis (Cvrčková et al., 2024), being, at the same time, a substrate of the vacuolar degradation pathway and a modulator of vacuole organization.

## 2 Materials and methods

### 2.1 Cloning

The AtFH1-mScarlet-I/TagBFP constructs were produced using a modified GoldenBraid cloning system (Sarrion-Perdigones et al., 2011) with an extended set of vectors (Dusek et al., 2020). In the first step, α13-Pink, α2-Basta resistance, and the matching stuffer fragments were ligated into the delta version of low-copy pLX plasmid carrying the BastaR selection marker (Pasin et al., 2017; Paz et al., 2006; Moravec et al., manuscript in preparation) using the BsmBI enzyme and a T4 ligase (Thermo Scientific, Waltham, Massachusetts, USA; see **Supplementary Table S1**).

For the expression of mScarlet-I- and TagBFP-labelled versions of AtFH1, the promoter (2,847 bp upstream from the start codon) and genomic sequence of *FH1* (At3g25500) were amplified in four separate PCRs using Q5 polymerase (NEB, Ipswich, Massachusetts, USA) to eliminate BsaI restriction sites (by introducing silent mutations) using FH1-delta primers (see **Supplementary Table S2**). Purified PCR products were, together with pUPD2 containing mScarlet-I (Bindels et al., 2017) or TagBFP (Subach et al., 2008) and pUPD1::ubiquitin terminator 3 (kindly provided by Matyáš Fendrych and Tomáš Moravec), inserted into the previously prepared pLX backbone in place of the pink cassette using the BsaI restriction enzyme and a T4 ligase.

### 2.2 Plant material

The *A. thaliana fh1-4* (SALK-N551065, Oulehlová et al., 2019) and *fh1-CRISPR* (Cifrová et al., 2020) mutant lines were published previously. The presence of the *fh1–4* allele was verified as described (Oulehlová et al., 2019), while *fh1-CRISPR* was detected by PCR amplification using the primers F_FH1_g4g7 and R_FH1_g4g7seq1 (**Supplementary Table S2**) and then verified by sequencing using the second primer.

Seeds from a VHP1:mGFP (Segami et al., 2014) expressing transgenic line were kindly provided by Falco Kruger and Melanie Krebs with approval of Shoji Segami and introduced into mutant backgrounds via crossing (see **Supplementary Table S3**).

The AtFH1-GFP construct described previously (Oulehlová et al., 2019) was independently transformed into the *fh1-CRISPR* background (see Cvrčková et al., 2024). For the AtFH1-mScarlet-I and TagBFP transgenes, verified plasmids were transformed into *Agrobacterium tumefaciens* and introduced into various backgrounds of *A. thaliana* using the floral dip method (Clough and Bent, 1998; see **Supplementary Table S3**). Unless stated otherwise, non-segregating transgenic lines were selected for further experiments from the progeny of selfed T1 plants. For quantitative measurements, a sister wild-type (WT) line of the mutant analyzed was used where possible; if such a line was not available, the parental WT genotype was used.

### 2.3 Growth conditions

Seedlings for imaging were grown *in vitro* on vertically positioned Petri dishes containing 1/2 Murashige and Skoog (MS) medium with 1.6% (w/v) agar and 1% (w/v) sucrose, pH-adjusted to 5.7, at 22°C with 16-h light/8-h dark cycle as described previously (Kollárová et al., 2025) for 5 days after sowing or transfer to the growth room, unless stated otherwise.

For the selection of transformants, the media were supplemented with corresponding antibiotics, with sucrose omitted, and the same *in vitro* growth conditions were used. Seedlings for seed propagation, crossing, or transformation were transferred to pellets (Jiffy, Zwijndrecht, Netherlands) and further grown under long-day conditions until seed ripening.

### 2.4 Pharmacological treatments

For Concanamycin A (ConcA) treatment, 4–5-day-old seedlings were treated with 0.5 μM ConcA (from 1 mM stock) or the same amount of DMSO (dimethyl sulfoxide, 0.05% v/v) for 24 hours. Wortmannin (WM) treatment was performed as described by Oulehlová et al. (2019) using 33.3 μM WM (diluted from 20 mM stock) in 1/2 liquid MS for 3 hours, with 0.165% DMSO (v/v) as a mock-treated control.

For SMIFH2 treatment, seeds were sterilized, stratified at 4°C for 2 days, and then sown directly on freshly prepared 1/2 MS plates containing either 20 μM SMIFH2 (diluted from 10 mM stock stored at −80°C for a maximum of a few months) or the same amount of DMSO (final concentration 0.2% v/v) and cultivated as described above.

LatB treatment was performed as described previously (Rosero et al., 2013) by transferring seedlings to 1/2 MS plates containing either 0.1 μM LatB (1 mM stock) or the same amount of DMSO (0.01% v/v) and cultivated as described above for approx. 6 or 24 hours prior to imaging.

### 2.5 Imaging

The microscopic images were acquired using spinning disc confocal microscopy (SDCM) with a Zeiss Axio Observer 7 microscope with a vertical stage equipped with a Yokogawa CSU-W1 spinning disk unit, alpha Plan-Apochromat 20×/0.8 or 100×/1.46 oil immersion objective, laser lines set at 488 or 561 nm, and PRIME 95B back-illuminated scientific CMOS (Complementary Metal–Oxide–Semiconductor) camera as described previously (Kollárová et al., 2021; Kočová et al., 2025). Alternatively, Nikon Ti-E was used, equipped with Yokogawa CSU-X1; laser box Omicron LightHUB ULTRA with laser lines set at 405, 488, and 561 nm; Plan-Apochromat 20×/0.75 or 100×/1.45 oil immersion objective; camera Photometrics Prime BSI; and back-illuminated scientific CMOS.

All images were processed using the Fiji software (Schindelin et al., 2012; RRID: SCR_002285). For the presentation, whole-field adjustments of brightness and contrast, rotation, cropping, and artificial coloring were performed.

### 2.6 Quantitative image data analyses

Tonoplast complexity was measured using both the established vacuolar morphology index (VMI; Löfke et al., 2015) and the novel tonoplast topology index (TTI) described in detail in Kočová et al. (2025). VMI values were logarithmically transformed using natural logarithm to bring the data distribution closer to normal (Kočová et al., 2025). Default filters and parameters were applied in the majority of cases. Typically, 10–20 atrichoblasts from eight or more roots per genotype or treatment were analyzed, with a minimum of six plants (except the tonoplast mobility determination in LatB-treated plants, where only four roots per treatment were used).

For determining tonoplast motility, a Fiji-based modification of the image-to-image correlation method previously employed by Vidali et al. (2010) to quantify cytoskeletal dynamics has been used. Briefly, single confocal plane video recordings were contrast-enhanced using the default Fiji Enhance Contrast function, converted to 8 bits, corrected for growth-dependent drift using the StackReg procedure with rigid body transformation (Thévenaz et al., 1998), and rotated to orient the root vertically. Subsequently, the rectangle tool was used to define five to 10 ROIs corresponding to individual cells per movie, and cell lengths (corresponding to the y dimension of these ROIs) were measured. For each ROI, the following procedure, automated using a recorded macro, was performed: 1) a cropped stack corresponding to the ROI (i.e., cell) was generated using the Duplicate function, 2) contrast was enhanced as above using the default procedure, and 3) the first frame of the sequence was duplicated, and the Image_CorrelationJ plugin (Chinga and Syverud, 2007; Chinga Carrasco, 2025) was used to determine the correlation coefficient (R) between the duplicated first frame as a fixed source and all frames of the movie as a sliding target, outputting average R values for each frame. Results were exported into Excel for subsequent processing.

When acquiring images and selecting individual cells for measurement, conscious effort was made to evaluate similarly sized cells in all samples of an experiment. After verifying that there were no statistically significant between-sample differences, cells were classified into “small” (below median length) and “large” (above median length) based on a median value obtained from the pool of all samples evaluated within the given experiment. In the case of odd cell numbers, the cell providing the median value was assigned to the group that contained its closest neighbor in terms of length.

### 2.7 Data processing and statistics

During the development of the analysis protocol and the generation of previews, data tables were processed using scripts written in Python language version 3.11.7 in the Jupyter notebook (RRID: SCR_018315) v 7.0.8 running under the Anaconda environment (RRID: SCR_025572) v 2.5.3. For datasheet handling, the Pandas library v 2.1.4 (McKinney, 2010; The Pandas development team, 2023; RRID: SCR_018214) was used, and Plotly (Plotly Technologies Inc, 2015; RRID: SCR_013991) was used to generate plots. The SciPy library v 1.11.4 (Virtanen et al., 2020; RRID: SCR_008058) and NumPy module components (Harris et al., 2020; RRID: SCR_008633) were used for statistics and correlation. In cases where analyzed quantitative data obviously or probably did not follow normal distribution (see Kočová et al., 2025), the Mann–Whitney test was used for evaluating statistical significance of differences. Multiplicity correction using the Benjamini–Hochberg FDR (false discovery rate) method was performed where necessary. In addition to the above-listed software tools, data were handled using MS Excel, and in some cases, online tools were employed for statistical calculations to perform Benjamini–Hochberg FDR correction (Radua et al., 2024; RRID: SCR_002554), ANOVA, the Kruskal–Wallis test, and the Mann–Whitney test (Vasavada, 2016), with practically identical results as with the above-listed tools obtained for a random subset of data analyzed using both methods. Box plots for final figures were generated using the R-based BoxPlotR tool (Spitzer et al., 2014; RRID: SCR_015629).

## 3 Results

### 3.1 AtFH1 enters the vacuole lumen upon transition into the root elongation zone

**Figure 1:**
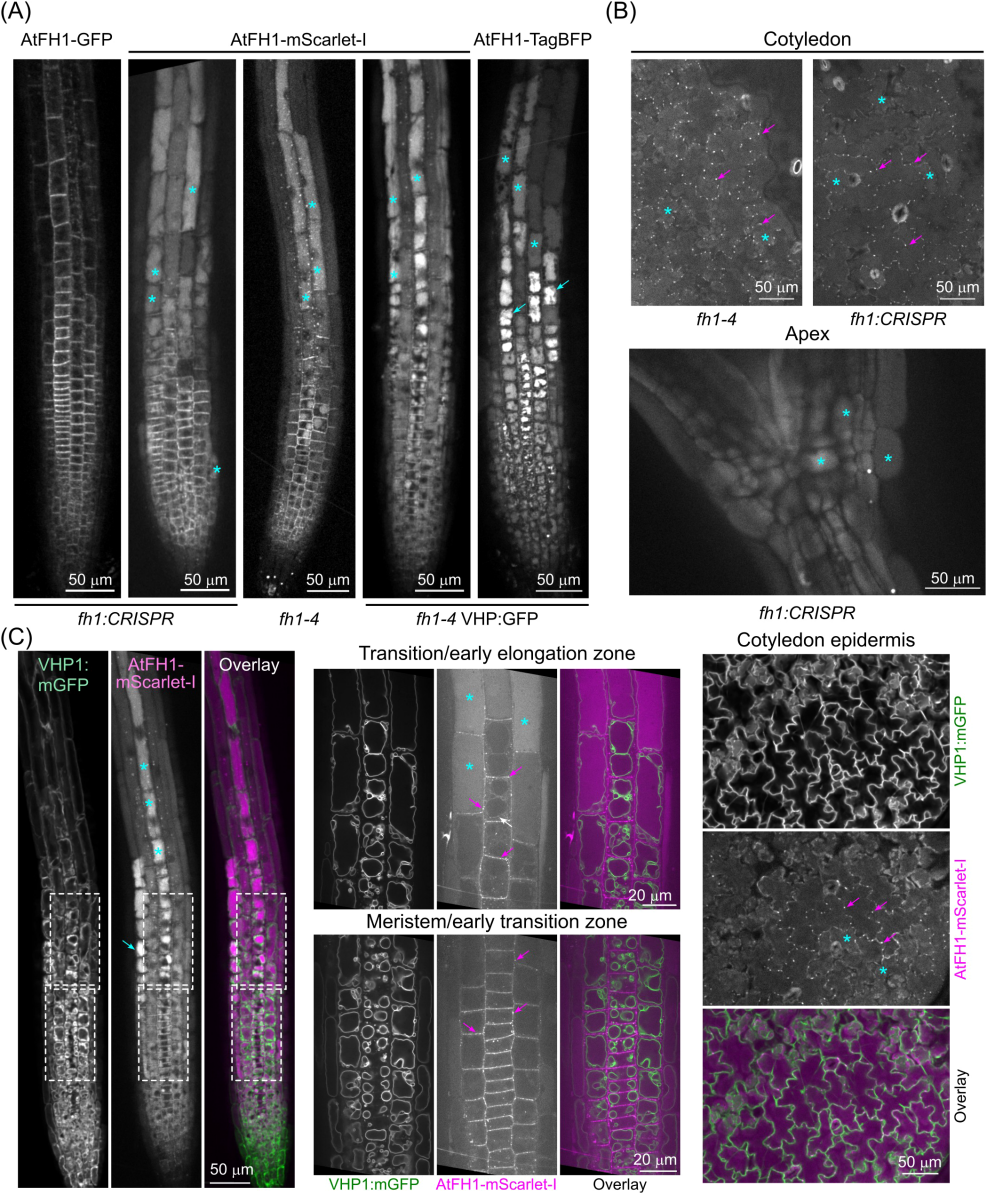
AtFH1 tagged with mScarlet-I or TagBFP entered the vacuole lumen. **(A)** Single confocal sections of root tips from 5–7-day-old seedlings grown under standard conditions, expressing the indicated fluorescent protein-tagged versions of AtFH1 in various genetic backgrounds (shown below the images). For seedlings expressing a second fluorescent marker, only the channel corresponding to the AtFH1-attached fluorochrome is shown. **(B)** Single confocal sections of cotyledon epidermis and apex from AtFH1-mScarlet-I-expressing seedlings of the indicated genotypes. **(C)** Colocalization of AtFH1-mScarlet-I and the tonoplast marker VHP1:mGFP in confocal sections from transgenic seedlings of the *fh1–4* background. Left – root tip overview, center – closeup of the areas of meristematic/early transition and transition/early elongation zones rhizodermis that are marked by dashed rectangles in the left panel, right - cotyledon epidermis. Examples of vacuolar lumen fluorescence are marked by cyan asterisks, possible vacuolar aggregates by cyan arrows, probable tonoplast-localized AtFH1 by a white arrow, and plasmodesmata by magenta arrows.

In order to compare the localization of AtFH1 tagged with the acidic pH-sensitive GFP label with versions labelled by other fluorochromes, we created transgenic plants expressing a less pH-sensitive mScarlet-I-labelled version of this formin from its native promoter, analogous to the previously described AtFH1-GFP lines (Oulehlová et al., 2019) except for using a different fluorochrome and linker and a promoter fragment that is 47 bp longer (see Materials and Methods). Microscopically, AtFH1-mScarlet-I showed plasma membrane, cytoplasmic puncta (likely endomembrane compartments—see Oulehlová et al., 2019), and plasmodesmata localization similar to that described previously for AtFH1-GFP (Oulehlová et al., 2019) in both root meristem and transition zones (**Figure 1A**) and above-ground tissues (**Figure 1B**). However, the punctate signal was stronger than that of AtFH1-GFP; in addition, vacuolar lumen signal was observed in some tissues, such as the root elongation and differentiation zone rhizodermis (**Figure 1A**), cotyledon epidermis, or various tissues of the hypocotyl (**Figure 1B**), and sometimes also in the lateral root cap or root meristematic zone, partially overlapping with the plasma membrane and plasmodesmata localization (**Figures 1A, B**). This localization pattern was observed in three independent transgenic lines of different genetic backgrounds, excluding the possibility of an artifact caused by accidental transgene rearrangement or insertional mutagenesis. A pattern similar to that of AtFH1-mScarlet-I was also found for AtFH1 fused to acidic pH-insensitive TagBFP (**Figure 1A**). The identity of the labelled compartment as the vacuole lumen was confirmed by simultaneous labelling with a tonoplast marker (**Figure 1C**).

Although the transient tonoplast signal of *AtFH1* is generally weak, bleaches rapidly, and is not always present, we were able to observe the tonoplast localization of AtFH1-mScarlet-I analogous to that observed previously for AtFH1-GFP (Oulehlová et al., 2019). Within individual rhizodermis cell files, the tonoplast signal always occurred rootward from the signal inside the vacuole lumen (**Figure 1C**), suggesting that the tonoplast and lumen localization of AtFH1 is partially separated in space and time, with tonoplast-localized formin likely moving into the vacuole lumen, possibly as a part of a degradation process. Consistent with this interpretation, we noticed that while the weak luminal signal of AtFH1-mScarlet-I was visible upon lower magnification also in the meristematic and early transition zones, it bleached very rapidly during subsequent observation and often disappeared within a few frames upon switching to a higher magnification (compare the left and central panels of **Figure 1C**; **Supplementary Figure S1**). Stronger luminal signal in the elongation zone, however, appeared more resistant to bleaching, probably due to the accumulation of a greater amount of the labelled protein. Remarkably, the vacuole lumen signal exhibited varying intensity among rhizodermis cell files and appeared to be stronger in the trichoblasts, which are readily distinguishable from the atrichoblasts by their more complex vacuome organization (**Figure 1C**).

**Figure 2:**
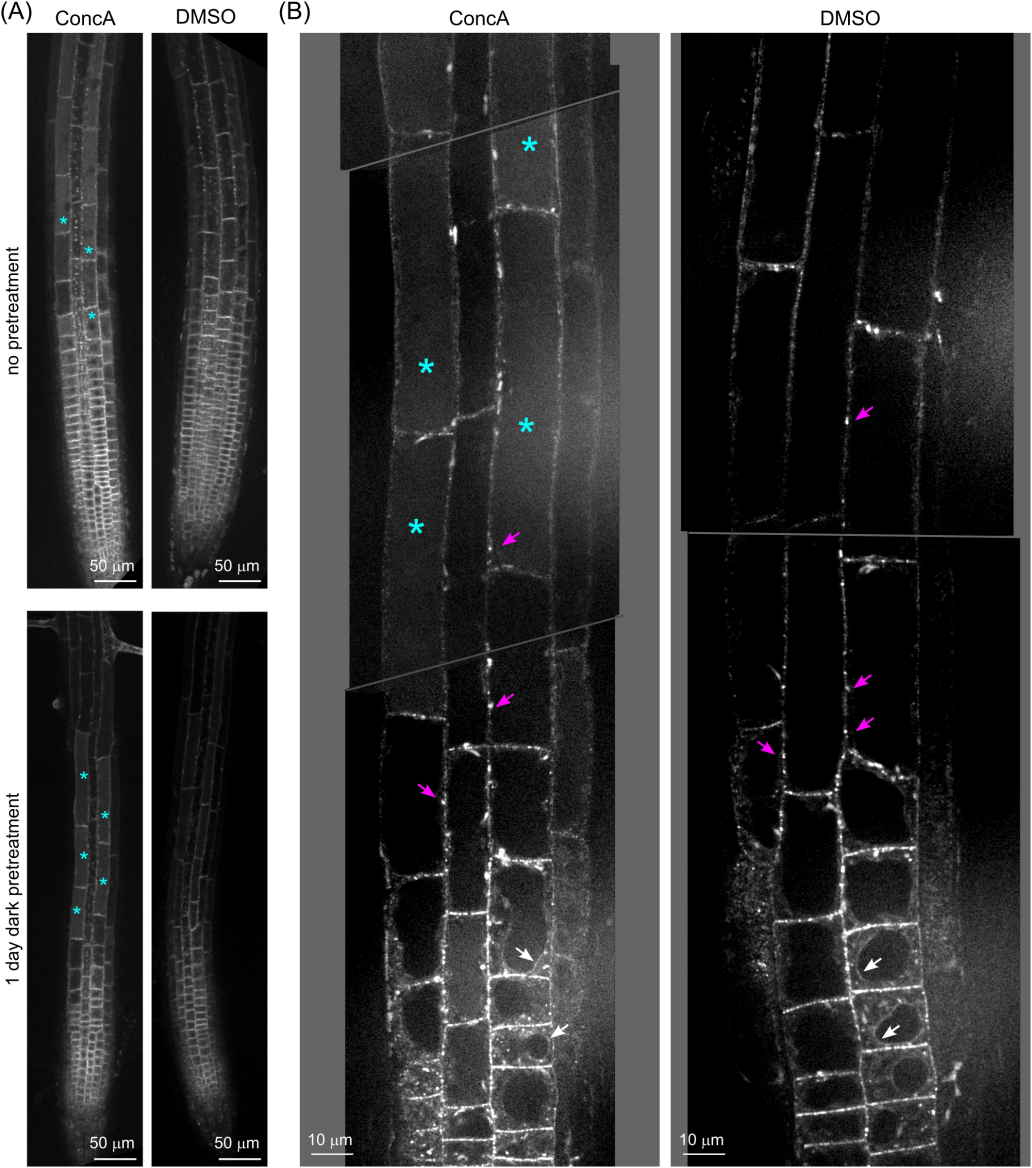
Concanamycin A treatment revealed AtFH1-GFP accumulation inside the vacuole. **(A)** Single confocal sections of root tips of 5–6-day-old *fh1:CRISPR* seedlings expressing AtFH1-GFP after 1 day of Concanamycin A (ConcA) treatment with or without 1 day of dark pretreatment. Control seedlings were mock-treated with DMSO. **(B)** Close-up of roots of *fh1:CRISPR* seedlings expressing AtFH1-GFP treated with ConcA after dark pretreatment as in panel A, compared to the control. Examples of vacuolar lumen fluorescence are marked by cyan asterisks, tonoplast-localized AtFH1 by white arrows, and plasmodesmata by magenta arrows.

The previously observed absence of AtFH1-GFP signal in the vacuole lumen (Oulehlová et al., 2019) is explained by the sensitivity of GFP to the vacuolar acidic pH. To investigate whether AtFH1-GFP is also targeted into the vacuole, we treated AtFH1-GFP plants with the vacuolar ATPase inhibitor ConcA, which retains the fluorescence of pH-sensitive GFP in the vacuole by increasing luminal pH (Tamura et al., 2003). Treatment with 0.5 μM ConcA for 24 hours in the dark (i.e., under conditions used in the Tamura et al., 2003, study) resulted in the appearance of GFP signal inside vacuoles in the root elongation zone (**Figure 2**), indicating that the absence of vacuolar AtFH1-GFP was indeed due to fluorochrome quenching by acidic conditions.

Thus, the level of the AtFH1 protein, whose gene is transcribed near-constitutively, i.e., expressed in the majority of vegetative tissues (see Oulehlová et al., 2019), appears to be regulated post-translationally by targeting excess AtFH1 to the vacuolar lumen for degradation.

### 3.2 AtFH1 localization is modulated by treatments affecting late endosomal and autophagy pathways

**Figure 3:**
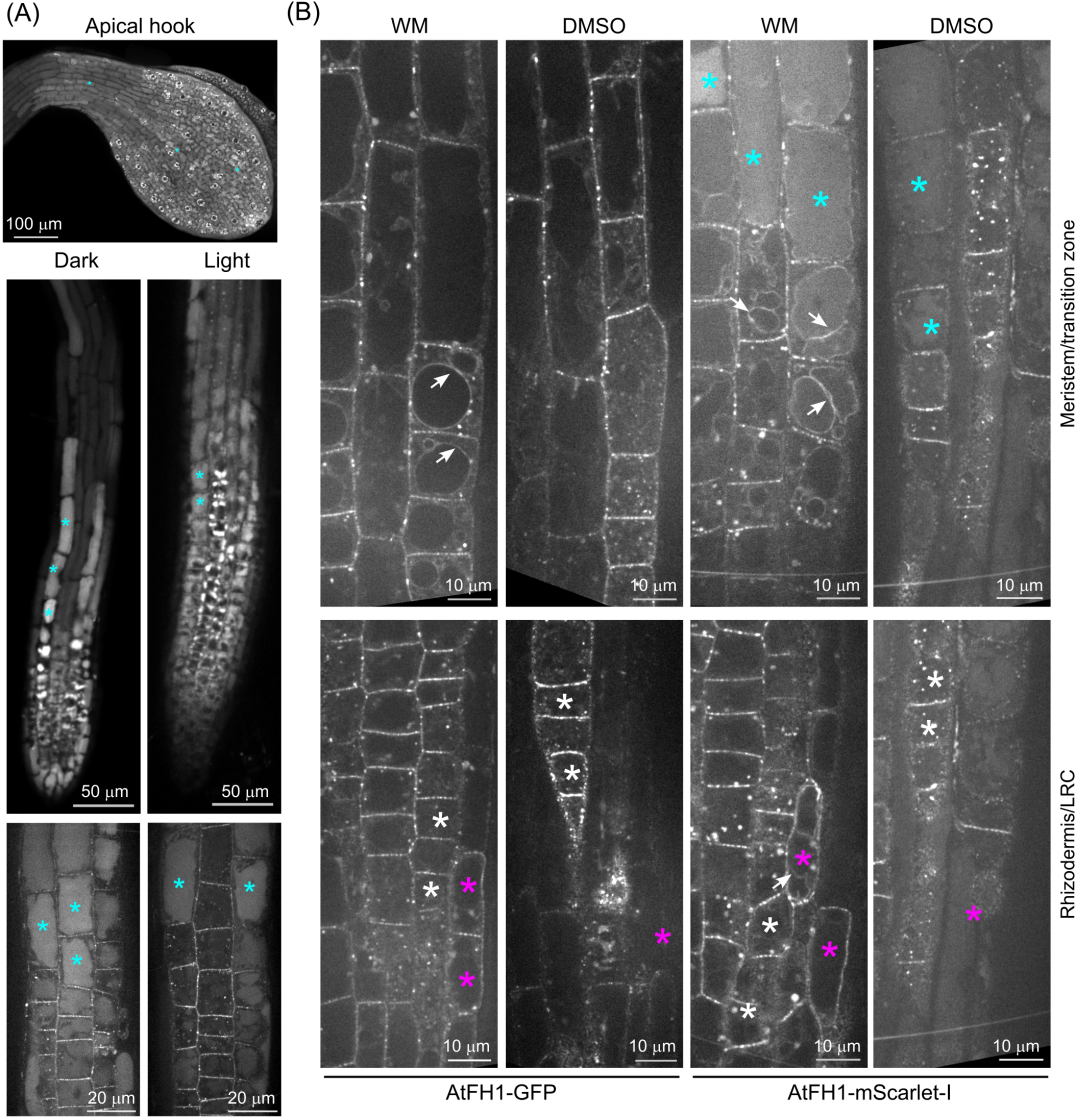
Effect of treatments affecting the autophagy pathway on vacuolar accumulation of fluorescent protein-tagged AtFH1. **(A)** Effect of etiolation on vacuolar AtFH1-mScarlet-I accumulation. A confocal Z-stack of the apical hook and single confocal sections of whole root tip and root meristematic/transition zones are shown. **(B)** Effect of wortmannin (WM) treatment on vacuolar and tonoplast localization of AtFH1-GFP or AtFH1-mScarlet-I in the root tip, shown alongside DMSO-treated controls. All images are single confocal sections from 5-day-old *fh1:CRISPR* background transgenic seedlings. Top row, transition and early elongation zone rhizodermis; bottom row, a section including both rhizodermis and the lateral root cap (LRC) in the meristematic zone. Examples of vacuolar lumen fluorescence are marked by cyan asterisks, LRC cells by magenta asterisks, rhizodermis cells by white asterisks, and tonoplast-localized AtFH1 by white arrows.

We therefore examined how treatments interfering with vacuolar protein delivery and degradation influence the localization and fate of fluorescently tagged AtFH1. While etiolation is known to trigger enhanced autophagy (Avin-Wittenberg et al., 2015), the pattern of AtFH1-mScarlet-I localization in etiolated seedlings did not noticeably differ from that of light-grown plants, aside from etiolated seedlings having shorter roots and therefore a different size of root developmental zones, and it exhibited an increased level of luminal signal in the meristematic zone (**Figure 3A**).

We next investigated the effects of the phosphatidylinositol 3- and 4-kinase inhibitor WM, known to interfere with late endosomal trafficking (causing late endosome/MVB dilation), as well as with autophagy and therefore also amphisome formation (Jaillais et al., 2008; Tse et al., 2004; Wang et al., 2009; Zhao et al., 2022). To our surprise, control (DMSO-treated) seedlings of all our transgenic lines, including the AtFH1-GFP-expressing *fh1–4* plants characterized in a previous report from our laboratory (Oulehlová et al., 2019), exhibited visible plasmalemma signal in the transition zone rhizodermis (**Figure 3B**, **Supplementary Figure S2**), which we reported as showing no plasmalemma fluorescence. We suspect that in our previous study (Oulehlová et al., 2019), we misidentified the cell layers; i.e., the alleged rhizodermis was actually the lateral root cap, and the alleged cortex was the rhizodermis. After treating AtFH1-GFP or AtFH1-mScarlet-I plants of both backgrounds by WM, we observed the appearance of bright fluorescent bodies and a major increase in fluorescent protein signal intensity in the plasmalemma of lateral root cap cells of WM-treated plants. These exhibited fluorescence intensity exceeding that commonly observed in atrichoblasts and comparable to that in trichoblasts, while in DMSO-treated controls, the lateral root cap was barely visible (**Figure 3B**, **Supplementary Figure S2**), confirming this suspicion. Overall, in the rhizodermis, WM treatment increased the amount and size of brightly fluorescent bodies (presumed enlarged endosomes) and enhanced tonoplast fluorescence. While we only sometimes observed tonoplast localization in DMSO-treated controls, similar to untreated seedlings, WM-treated plants exhibited tonoplast labelling in nearly all the cells of the transition and early elongation zones, and sometimes in the lateral root cap cells. In the case of AtFH1-mScarlet-I, we sometimes observed the tonoplast localization of vacuoles exhibiting luminal fluorescence (**Figure 3B**). These observations strongly indicate that AtFH1 delivery to the vacuole is dependent on late endosomal and possibly also autophagy pathways.

### 3.3 Loss of *FH1* function or pharmacological formin inhibition alters vacuole organization

**Figure 4:**
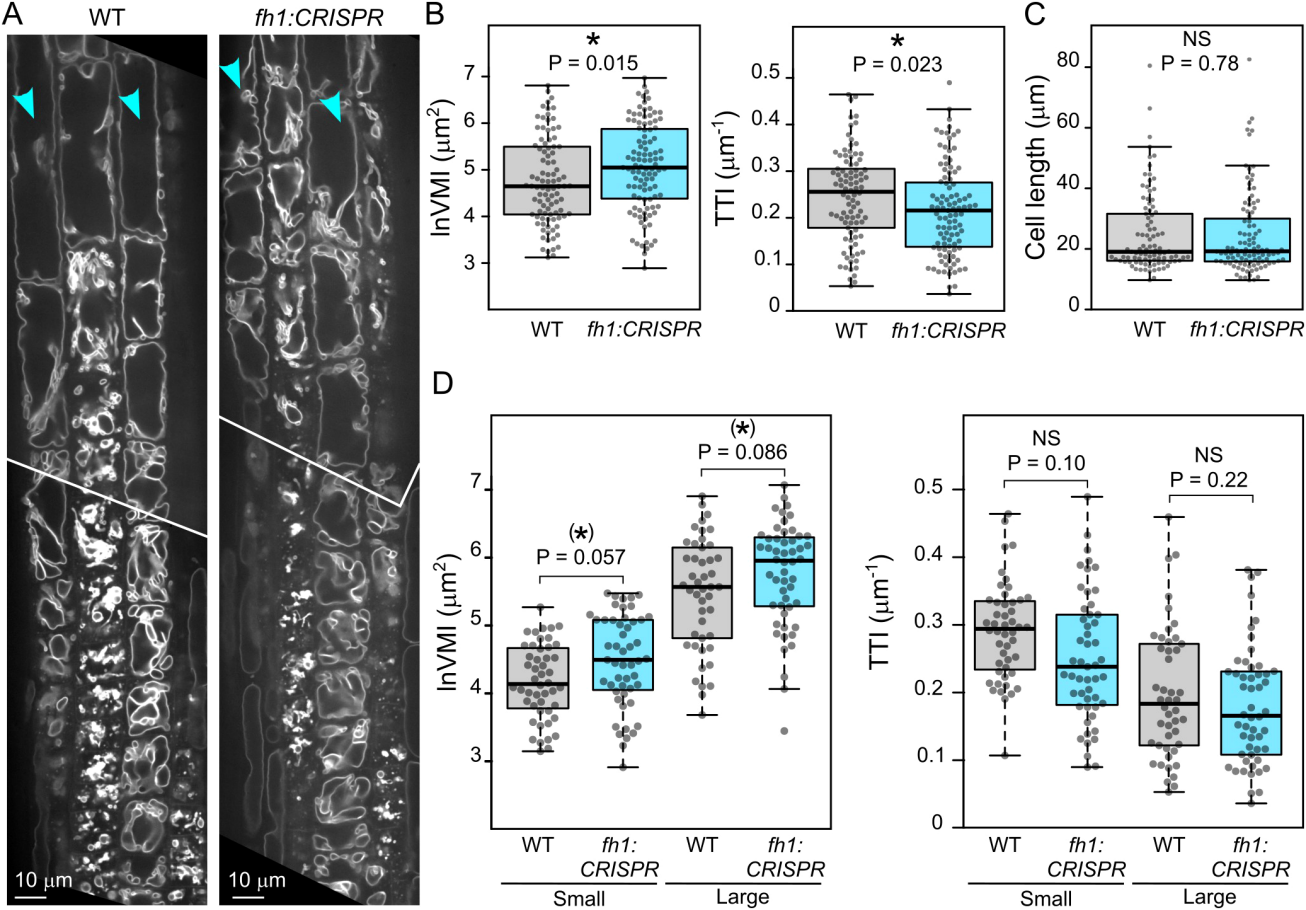
Comparison of vacuole organization in wild-type (WT) and *fh1:CRISPR* seedling roots. **(A)** Representative optical sections of root tips of transgenic plants expressing the tonoplast marker VHP1:mGFP in WT and *fh1:CRISPR* genetic background. **(B)** Quantitative characterization of tonoplast organization in atrichoblasts of WT and *fh1:CRISPR* plants expressing VHP1:mGFP, as determined by two metrics: the vacuolar morphology index (VMI; left) and the tonoplast topology index (TTI; right). Only roots growing on the surface of the agar medium were evaluated. **(C)** Comparison of cell length distribution of the WT and mutant atrichoblasts evaluated in panel B, documenting comparable cell size in both samples. **(D)** Quantitative characterization of tonoplast organization in atrichoblasts, as in panel B, with cells split into two size categories: small (below median length) and large (above median length). Significance of between-genotype differences in panels B–D is documented using Mann–Whitney p-values; (D) corrected for multiplicity using the Benjamini–Hochberg method. Significance of differences is indicated by * for (0.01 < p ≤ 0.05), (*) for (0.05 < p ≤ 0.1).

We next employed transgenic plants expressing the tonoplast marker VHP1:mGFP in WT and loss-of-function *fh1* mutant backgrounds to study the possible involvement of AtFH1 in central vacuole development. Since there was no readily noticeable (i.e., qualitative) difference in tonoplast organization between WT and mutant plants (**Figure 4A**), we employed two complementary quantitative metrics to capture possible differences in vacuome configuration: the well-established VMI, which reflects the dimension of the largest vacuolar compartment on an optical section of a cell (Löfke et al., 2015), and our newly developed TTI, which also takes into account tonoplast convolutedness by counting tonoplast crossing per unit length of a diagonal cell transect (Kočová et al., 2025).

In two independent mutant backgrounds, both metrics indicated less complex central vacuole structure, i.e., morphologically more advanced central vacuole consolidation, in rhizodermis atrichoblasts of *fh1* mutants compared to those of WT plants, as indicated by higher VMI values (corresponding to bigger nascent vacuolar compartments) and lower TTI values (reflecting fewer membrane crossings by a diagonal cell transect) (**Figure 4B**, **Supplementary Figure S3A**). To eliminate the effects of possible differences in atrichoblast developmental stages among genotypes, we analyzed cells of comparable size in mutant and WT plants (**Figure 4C**, **Supplementary Figure S3B**). This also allowed us to further examine the effects of cell size (and developmental stage) by separately analyzing small cells (defined as those below median cell length) and large ones (above median cell length). Although the observed between-genotype differences were not always statistically significant, qualitatively similar results were obtained consistently in independent biological replicates for different alleles and genetic backgrounds (**Figure 4D**, **Supplementary Figure S3C**). This suggests a trend of greater differences occurring in smaller cells, again consistent with the central vacuole maturing earlier, i.e., closer to the root tip, in the mutants.

**Figure 5:**
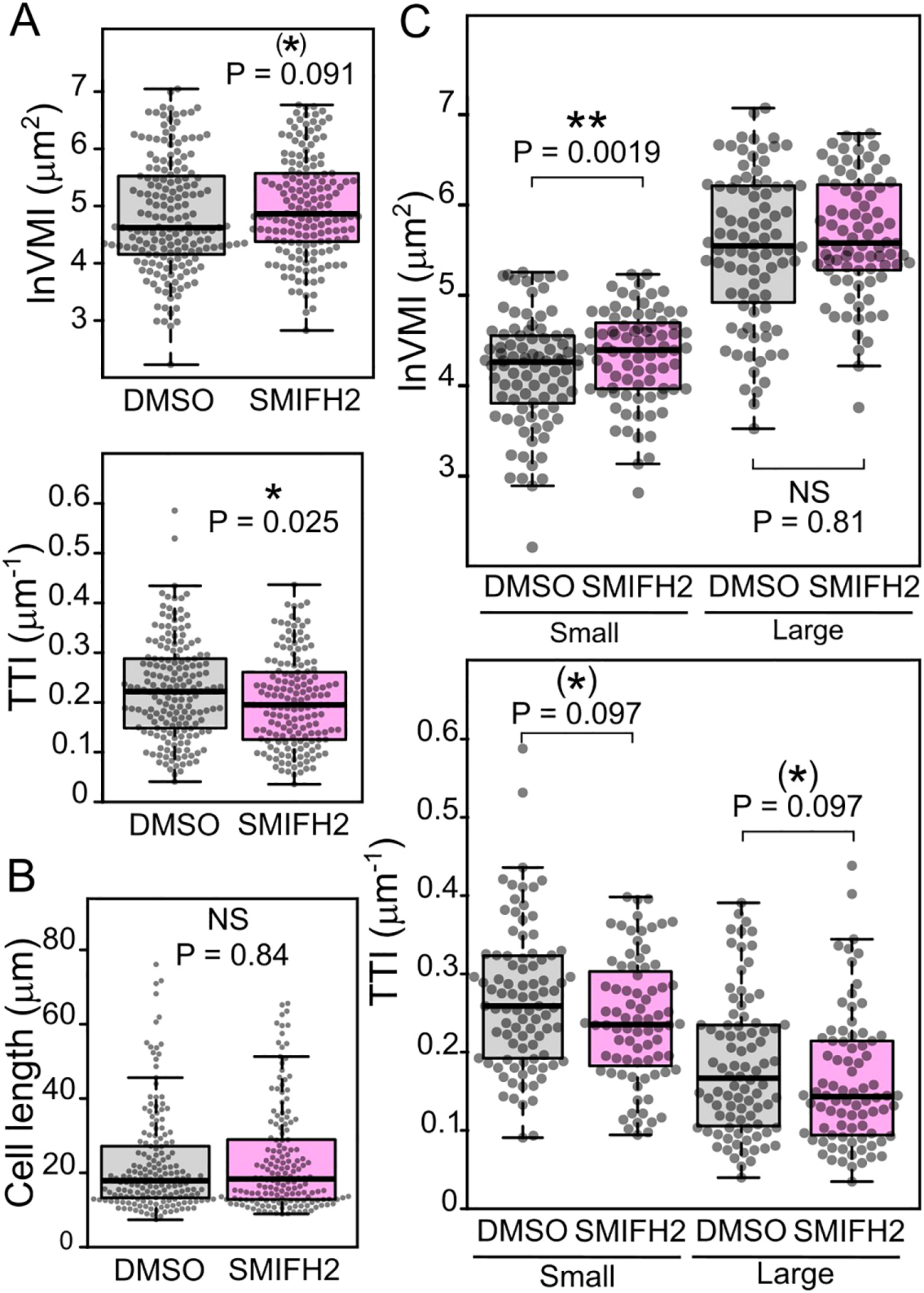
Effects of formin inhibition on atrichoblast vacuole organization. **(A)** Quantitative characterization of tonoplast organization in atrichoblasts of wild-type (WT) plants expressing the tonoplast marker VHP1:mGFP (sister segregants of the *fh1:CRISPR* mutant), grown on control (DMSO-containing) or SMIFH2-containing media, as determined by the vacuolar morphology index (VMI) and tonoplast topology index (TTI) metrics. **(B)** Comparison of cell length distribution of the WT and mutant atrichoblasts evaluated in panel A, documenting comparable cell size in both samples. **(C)** Quantitative characterization of tonoplast organization in atrichoblasts, as in panel A, with cells split into two size categories: small (below median length) and large (above median length). Significance of between-genotype differences is documented using Mann–Whitney p-values; (C) corrected for multiplicity using the Benjamini–Hochberg method. Significance of differences is indicated by ** for p ≤ 0.01, * for 0.01 < p ≤ 0.05, (*) for 0.05 < p ≤ 0.1.

Treatment with the formin FH2 domain inhibitor SMIFH2 (Rizvi et al., 2009) qualitatively reproduced the effects of the loss-of-function *fh1* mutations in two different WT genetic backgrounds, i.e., caused an increase in VMI and a decrease in TTI, with at least one of the metrics documenting a statistically significant effect (**Figure 5A**, **Supplementary Figure S4A**). Since the size of analyzed cells was comparable in treated and untreated plants (**Figure 5B**, **Supplementary Figure S4B**), we could again examine separately the effects of formin inhibition in small and large cells, with the results suggesting that the impact of formin inhibition is notable especially in small cells (**Figure 5C**, **Supplementary Figure S4C**).

**Figure 6:**
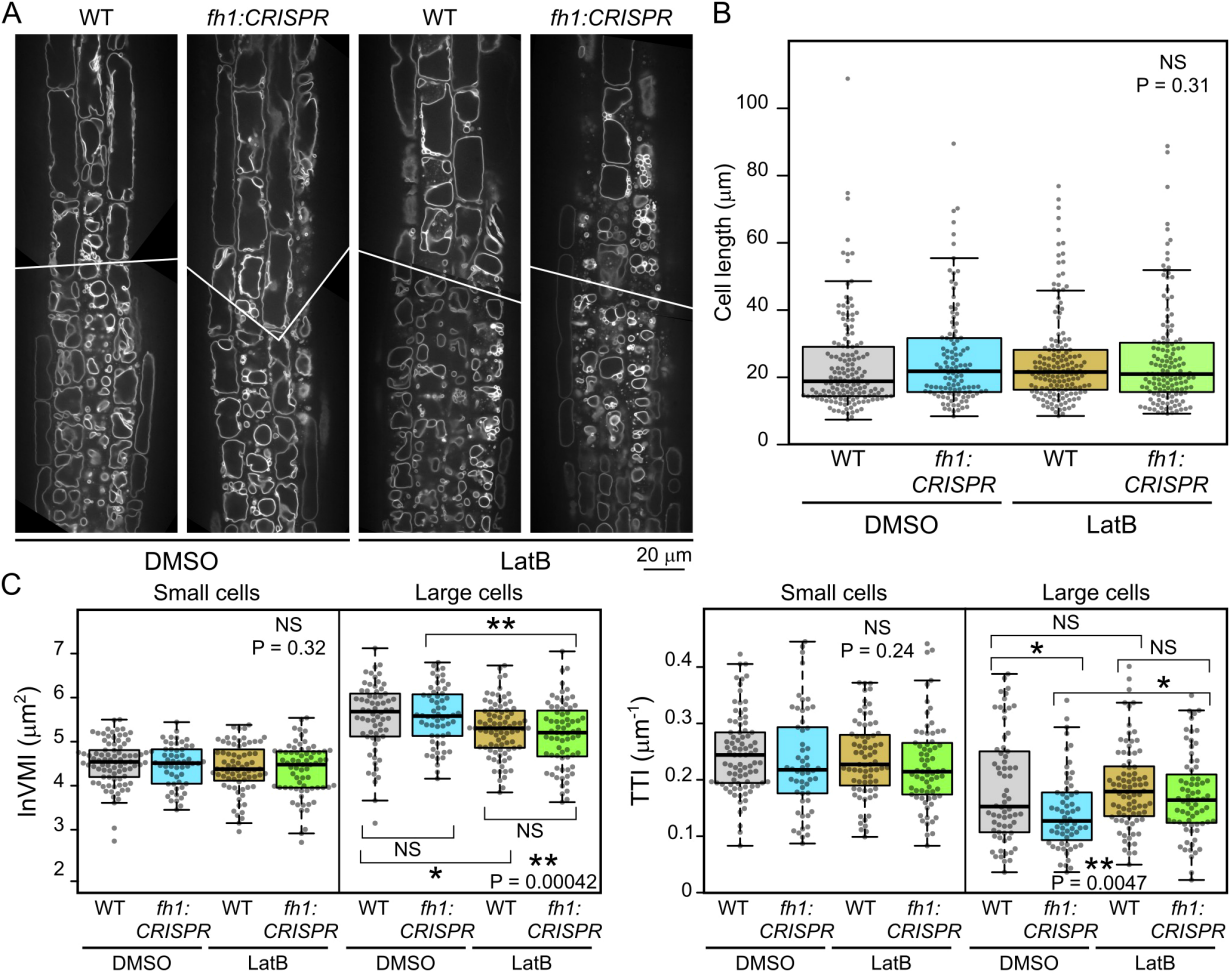
Effects of actin disruption on atrichoblast vacuole organization in wild-type (WT) and *fh1:CRISPR* plants. **(A)** Representative optical sections of root tips of transgenic plants expressing the tonoplast marker VHP1:mGFP in WT and *fh1:CRISPR* genetic background after 24 hours of Latrunculin B (LatB) treatment compared to DMSO-treated controls. **(B)** Comparison of cell length distribution of atrichoblasts of the indicated genotype and treatment combinations that were subjected to quantitative analysis of tonoplast organization, documenting comparable cell size in all samples. **(C)** Quantitative characterization of tonoplast organization in atrichoblasts, as in panel B, with cells split into two size categories: small (below median length) and large (above median length). Significance of between-sample differences (or lack thereof) is documented in panels B and C by Kruskal–Wallis omnibus null hypothesis p-values (determined separately for small and large cells in panel C). In panel C, asterisks reflect p-values for the indicated differences as obtained by Dunn’s procedure and corrected for multiplicity using the Benjamini–Hochberg method (** for p ≤ 0.01; * for 0.01 < p ≤ 0.05; NS for p ≥ 0.1).

Since *fh1* mutants were previously documented to exhibit altered LatB sensitivity (Rosero et al., 2013, 2016), we examined how prolonged treatment with the actin polymerization inhibitor LatB at doses previously shown to cause reduction of root growth rate and subtle developmental alterations without impairing seedling survival (Rosero et al., 2013, 2016) influences the effect of *fh1* mutations on vacuole organization and development. Upon treatment of seedlings expressing the VHP1:mGFP tonoplast marker in either WT or *fh1:CRISPR* background with this inhibitor, the overall tonoplast structure of LatB-treated roots changed noticeably compared to DMSO-treated control, with effects observable after several hours of treatment. Generally, small vacuolar compartments were inflated, while the fusion of small compartments into one big vacuole was impaired in larger cells (**Figure 6A**), matching previously described observations (e.g., Scheuring et al., 2016; Oulehlová et al., 2019). In some roots, trichoblasts and atrichoblasts even became nearly indistinguishable (except for slightly stronger VHP1:mGFP signal in the former). Quantitative analysis confirmed the previously observed trend toward more advanced central vacuole development (higher VMI and lower TTI) in *fh1* mutants both in the control and after LatB treatment, although not all of the observed differences were statistically significant. The inhibitor had qualitatively similar effects in WT and mutant plants; i.e., especially large cells displayed decreased VMI and increased TTI after LatB treatment, indicating higher vacuole shape complexity, compared to mock-treated controls (**Figures 6B, C**).

### 3.4 Loss of *FH1* reduces tonoplast motility

**Figure 7:**
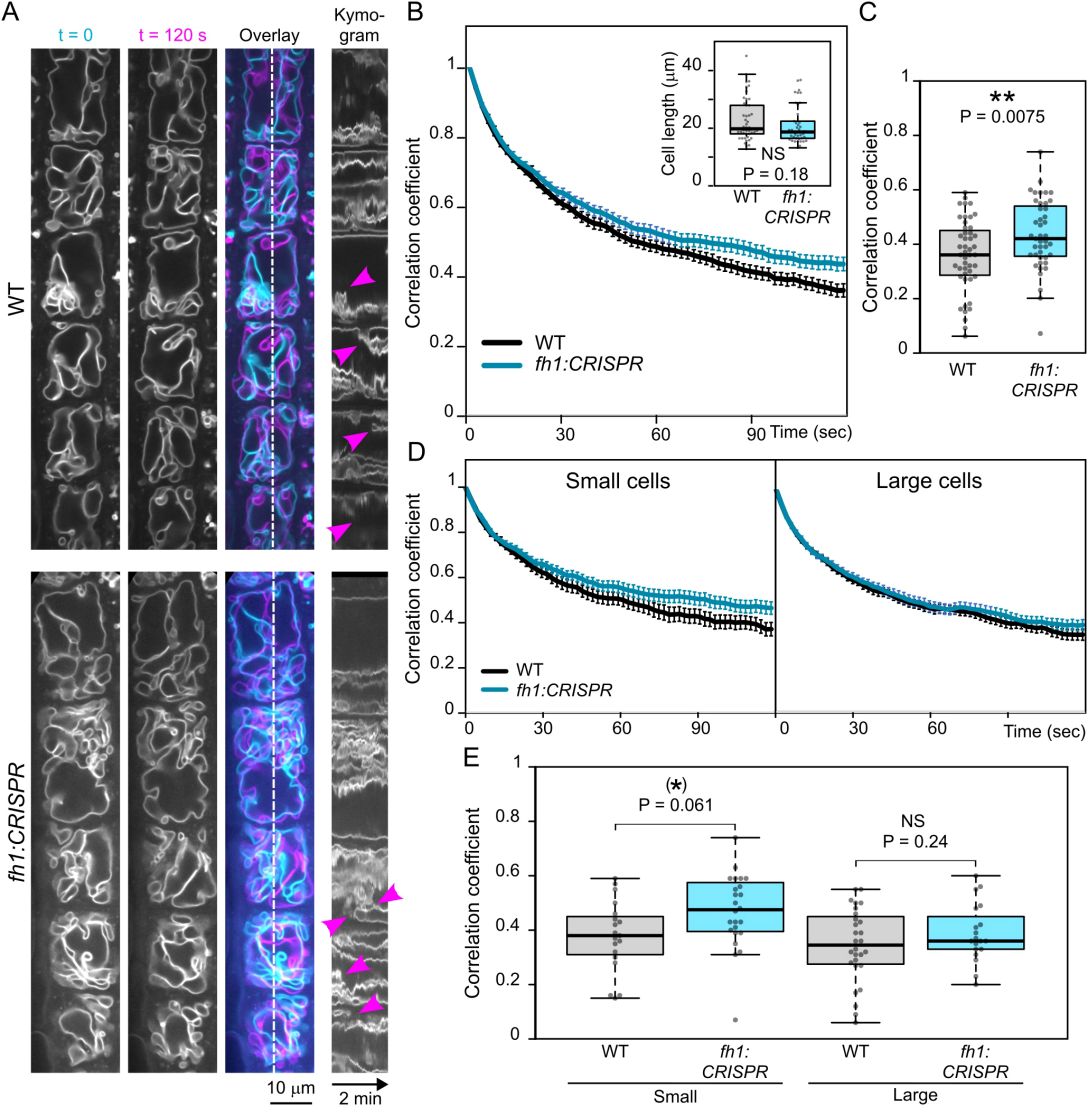
Comparison of tonoplast motility in wild-type (WT) and *fh1:CRISPR* atrichoblasts. **(A)** Two frames taken 2 minutes apart from a video recording of transgenic seedlings expressing the tonoplast marker VHP1:mGFP in WT and *fh1:CRISPR* genetic background, shown together with their superposition (cyan, t = 0; magenta, t = 120 sec) and a kymogram taken along a longitudinal transect indicated by the dashed line. Magenta arrowheads denote rapid events. **(B)** Average frame to first frame pixel intensity correlation coefficients (from at least 40 cells per genotype and time point), plotted against time. Error bars denote SEM. Inset: cell length distribution of the analyzed cells, documenting comparable cell size in both samples. Only roots growing on the surface of the agar medium have been evaluated. **(C)** Distribution of average single-cell pixel intensity correlation coefficients among two frames taken 120 seconds apart for each genotype, i.e., corresponding to the rightmost end of plots in panel B. **(D)** Average frame to first frame pixel intensity correlation coefficients, plotted as in panel B, separately for small (below median length) and large (above median length) cells. **(E)** Distribution of average single-cell pixel intensity correlation coefficients among two frames taken 120 seconds apart, plotted as in panel C, separately for small and large cells. Significance of between-genotype differences or lack thereof is documented using the Mann–Whitney p-value in panel B and by t-test p-values in panels C and E; **(E)** corrected for multiplicity using the Benjamini–Hochberg method. Significance of differences is indicated by ** for p ≤ 0.01, (*) for 0.05 < p ≤ 0.1.

The above-described developmental changes in tonoplast organization represent a relatively slow ontogenetic process. We subsequently also examined the impact of *fh1* loss-of-function mutations on rapid, cytoplasmic streaming-related tonoplast motility. While there was no dramatic, visually detectable qualitative effect (**Figure 7A**, **Video S1**, **Video S2**), quantitative analyses revealed significantly slower tonoplast dynamics in *fh1:CRISPR* mutants (**Figures 7B, C**), with the difference being due mainly to the contribution of smaller cells (**Figures 7D, E**), similar to the effect of *fh1* mutations on vacuole organization. A qualitatively similar overall effect, although weaker, was also found for the second mutant allele, *fh1-4* (**Supplementary Figure S5**).

**Figure 8:**
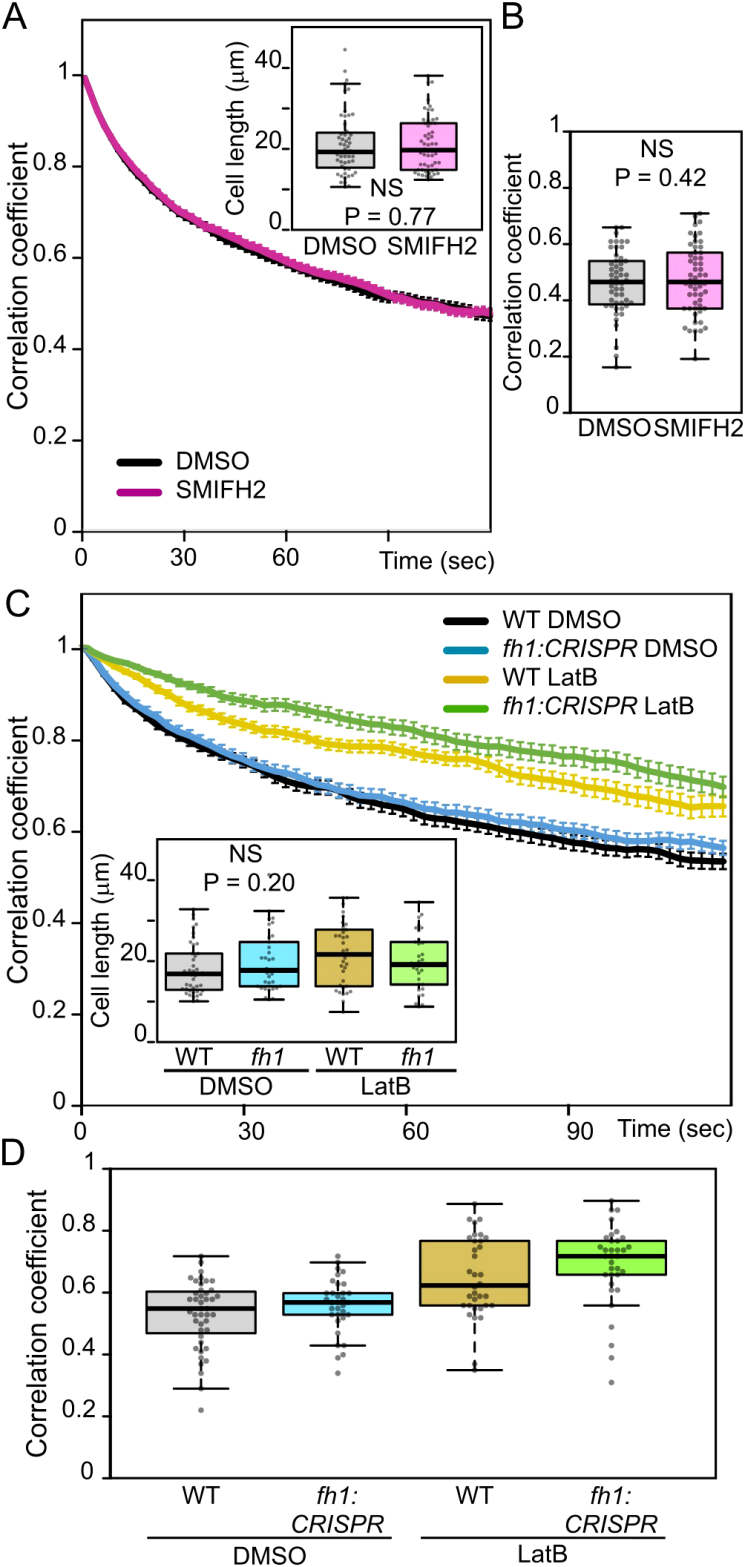
Effects of pharmacological treatments on atrichoblast tonoplast motility. **(A)** Average frame to first frame pixel intensity correlation coefficients from videos of control (DMSO-treated) and SMIFH2-treated wild-type (WT) seedlings congenic to *fh1-4*, plotted against time. The recordings were taken from a subset of seedlings evaluated for **Figure 5B**. Error bars denote SEM. Inset: cell length distribution of the analyzed cells, documenting comparable cell size in both samples. **(B)** Distribution of average single-cell pixel intensity correlation coefficients among two frames taken 120 seconds apart for cells analyzed in panel A, i.e., corresponding to the rightmost end of plots in panel A. **(C)** Average frame to first frame pixel intensity correlation coefficients from videos of WT and *fh1:CRISPR* seedlings mock-treated with DMSO or Latrunculin B (LatB) for 6 hours, plotted against time. Error bars denote SEM. Inset: cell length distribution of the analyzed cells, documenting comparable cell size in all samples. **(D)** Distribution of average single-cell pixel intensity correlation coefficients among two frames taken 120 seconds apart for cells analyzed in panel C, i.e., corresponding to the rightmost end of plots in panel C. Significance of between-sample differences or lack thereof is documented using the Mann–Whitney p-value in panel A, t-test p-value in panel B, and Kruskal–Wallis p-value in panel C. **(D)** Two-way ANOVA identified significant effect of both genotype (p = 0.026) and treatment (p < 0.0001), but not their interaction (p = 1).

Surprisingly, prolonged treatment with SMIFH2, which elicited vacuolar organization changes in the same individual plants, did not affect rapid tonoplast motility (**Figures 8A, B**). This suggests different mechanisms of AtFH1 participation in ontogenetic vacuole configuration and in rapid cytoplasmic movements. Both processes, however, involve the actin cytoskeleton since rapid tonoplast movements were already inhibited by relatively short LatB treatment to a similar extent in WT and *fh1:CRISPR* plants (**Figures 8C, D**).

## 4 Discussion

In this report, we are addressing the fate and function of the *Arabidopsis* Class I transmembrane formin AtFH1 in developing root tissues. The *FH1* (*AtFH1*) gene (At3g25500) is transcribed in the majority of tissues (Hruz et al., 2008), suggesting a possible “housekeeping” function. However, a previous study from our laboratory (Oulehlová et al., 2019) revealed that GFP-tagged AtFH1 expressed under the control of its native promoter can be detected in the root meristem and some above-ground tissues, but disappears in the root elongation zone. Thus, the pattern of AtFH1 protein expression does not match that of its gene transcription, suggesting post-transcriptional and possibly post-translational regulation, which has been documented in many cases in plants (reviewed in Petrillo et al., 2014; Agbemafle et al., 2023). GFP-tagged AtFH1 changes its localization pattern within the root tip, from dominant plasma membrane localization in the meristematic zone to transient tonoplast localization in the transition zone, before the protein ultimately disappears in the elongation and later zones; mobile endomembrane compartments of likely endosomal characters are also labelled in all zones showing AtFH1-GFP fluorescence (Oulehlová et al., 2019). This pattern suggests that the observed tonoplast localization may indicate vacuolar degradation of AtFH1, and the present study aimed to test this hypothesis while also exploring the possible role of AtFH1 in vacuole organization and dynamics.

Ubiquitin-dependent proteasomal, late endosomal, or autophagic protein degradation is well documented in plants, although not all these pathways are well characterized (see Sieńko et al., 2020). As plasmalemma proteins, plant formins are believed to undergo controlled degradation primarily through the ubiquitin–proteasome system involving E3 ubiquitin ligases (Isono et al., 2024). Plant membrane protein degradation via autophagy is not as well documented or characterized as degradation by the ubiquitin–proteasome pathway. However, (macro)autophagy in plants exists as a general intracellular degradation mechanism that sequesters cytoplasmic components into autophagosomes that fuse with vacuoles for degradation and recycling. In addition, many transmembrane proteins localized to the plasma membrane, including several transporters (PIN, BOR1, and IRT1), which were found to be sorted into the MVBs through the ESCRT microautophagy-like pathway (reviewed by Paez Valencia et al., 2016; Isono and Kalinowska, 2017; Sieńko et al., 2020; Yang et al., 2021), with subsequent fusion of MVBs with the tonoplast, the internalization of tonoplast-derived membrane vesicles into the vacuole lumen, and cargo release for degradation by a mechanism not yet characterized in plants but possibly analogous to processes previously described in yeast (Yagyu and Yoshimoto, 2024; Kagohashi et al., 2023). Degradation inside the vacuole lumen, either by an MVB-mediated pathway or a direct tonoplast vesicle retrieval into the vacuole that could be considered a form of microautophagy, has been documented also for the tonoplast-resident potassium channel TPK1 (Maîtrejean et al., 2011; Maîtrejean and Vitale, 2011), although in none of the so far described plant cases was a plasmalemma protein undergoing degradation found to dwell, even transiently, on the tonoplast, in contrast to AtFH1. Our observations, therefore, suggest the involvement of an autophagy-related pathway in AtFH1 delivery and degradation in the vacuole. Additional experiments involving colocalization with markers of distinct late endosomal/MVB and autophagy-related compartments, such as ATG8 for macroautophagy (Yang et al., 2021), ARA7 or ESCRT for the late endosome/MVB pathway (Minamino and Ueda, 2019; Sieńko et al., 2020), or mutant effect studies, will be required to identify the specific processes and mechanisms.

While GFP and its enhanced variant eGFP (enhanced GFP) remain the most popular fluorescent proteins, one of their major drawbacks is the absence of signal in acidic compartments due to rapid pH-dependent quenching and, in light-grown plants (like those used in our experiments), also degradation (Tamura et al., 2003; Roberts et al., 2016). The possible presence of AtFH1-GFP in the vacuole could thus have been missed in previous experiments (Oulehlová et al., 2019) due to this effect. Several less pH-sensitive fluorescent proteins are currently available, including a pH-insensitive variant of GFP (Roberts et al., 2016), the red fluorescent protein mCherry with pKa of 4.5, or mScarlet variants with *in vitro* pKa of approximately 5.3–5.4 (Bindels et al., 2017), with even more acidic values measured *in vivo* (Botman et al., 2019), in contrast to pKa ~ 6 of eGFP (Haupts et al., 1998). We thus constructed an alternative fluorescent version of AtFH1 using mScarlet-I, which exhibited fluorescence in the vacuolar lumen of cells in the transition and elongation zones. Similar localization was also found for the blue fluorescent protein TagBFP (Subach et al., 2008), which, with its pKa of 2.7, is supposed to be more pH resistant than any of the abovementioned fluorescent proteins. The observed luminal fluorescent protein signal may represent either a (complete or partial) fusion protein or a free fluorochrome released into the lumen (possibly after late endosome/MVB, amphisome, or microautophagic membrane internalization and degradation of the formin part of the fusion protein) and persisting for some time within the vacuole. The increased punctate cytoplasmic staining in the elongation zone rhizodermis of plants expressing AtFH-mScarlet-I, compared to AtFH1-GFP, most likely corresponding to endosomes (see Oulehlová et al., 2019), i.e., compartments with acidic luminal pH (Shen et al., 2013), also corroborates our interpretation.

Our observations indicate a difference in the behavior of the vacuolar lumen mScarlet-I signal among root developmental zones. While the fluorescence is relatively stable in the root elongation zone, rapidly bleaching “aggregate-like” structures appear in the meristematic zone. Since the vacuolar pH is higher in the elongation zone compared to the meristematic zone (Bassil et al., 2011; Kriegel et al., 2015), we suspect that the rapid bleaching in younger tissues reflects a combination of more acidic pH closer to pKa of mScarlet-I with strong laser illumination upon higher magnification, resulting in rapid loss of mScarlet-I fluorescence. Also, the recent *in vivo* characterization of fluorescent proteins revealed a low Hill’s coefficient in the case of mScarlet proteins, another important parameter, whose lower value represents higher pH sensitivity (Botman et al., 2019), although there may be differences between plant and yeast systems.

The loss of GFP fluorescence in acidic pH can be partially prevented by pharmacological treatments such as the inhibition of the vacuolar ATPase with ConcA or by keeping the sample in the dark prior to imaging since low pH combined with blue light leads to the rapid degradation of GFP (Tamura et al., 2003). ConcA treatment, sometimes with varying conditions of application, was used to visualize GFP inside the vacuole, also for other reported autophagically degraded GFP-tagged membrane proteins, such as PIN2 (Kleine-Vehn et al., 2008) or BOR1 (Takano et al., 2005). Indeed, treatment of our AtFH1-GFP seedlings with ConcA using the protocol from Tamura et al. (2003) induced green fluorescence inside elongation zone vacuoles but not in the meristem. This further supports the hypothesis that AtFH1 is targeted for vacuolar degradation depending on the cells’ developmental status, although the more acidic pH of the meristematic cell vacuome (Bassil et al., 2011) may also make the ConcA effect less prominent there.

However, dark treatment or observation of roots of etiolated seedlings did not have any visible effect on AtFH1-GFP pattern, suggesting that additional parameters, such as expression level or some specific characteristics of the protein, may play some role. It is worth noting that in our preliminary experiments, at least 5 hours of ConcA treatment was needed for the AtFH1-GFP signal to appear in the vacuolar lumen, while merely moving the seedlings to the dark for 1–2 days for vacuole-targeted free GFP (Tamura et al., 2003) or 3 hours (but not 2 hours) of treatment with ConcA at a concentration comparable to ours for the BOR1 boron transporter under conditions of boron excess where it is rapidly degraded (Takano et al., 2005) was reported to have an observable effect for these target proteins. This highlights the contribution of target protein-specific trafficking and degradation dynamics that would perhaps deserve further attention. Another *Arabidopsis* class formin, AtFH5, was found to localize in the vacuolar lumen after 6 hours of combined treatment with ConcA and the autophagy inducer AZD8055, but not after the application of each drug separately (Kollárová et al., 2025), suggesting that vacuolar degradation may be a common feature of plant Class I formins, in contrast to the current view of preferred proteasome degradation (see Isono et al., 2024).

Membrane trafficking of AtFH1 to the vacuole was blocked by WM, an inhibitor of phosphatidylinositol-3-kinase (PI3K), causing enlargement of MVB/late endosomes/prevacuolar compartments (Kleine-Vehn et al., 2008; Wang et al., 2009). We did indeed observe some enlarged compartments in WM-treated plants expressing fluorescently tagged AtFH1, but the signal also accumulated at the tonoplast, suggesting that the internalization of tonoplast-localized AtFH1 was impaired more than its transport to the tonoplast itself. An analogous phenomenon was observed during microautophagy in the yeast *Pichia pastoris*, where WM in some situations inhibited tonoplast invagination, i.e., an early step of cargo internalization into the vacuole (Sakai et al., 1998). Our observations suggest that AtFH1 expression is also regulated at the protein level by sequestering excess AtFH1 into the vacuolar lumen for degradation. However, further experimental study (as suggested above) would be needed to ascertain whether this process involves the MVBs, amphisomes (i.e., macroautophagosomes fused with MVBs), direct microautophagy from tonoplast invaginations, or another pathway.

Since Class I formins are integral membrane proteins with possibly multiple cytoplasmic interaction partners, this raises the question of whether AtFH1 could also participate in the shaping of the tonoplast membrane, acting thus as an “active cargo” of membrane trafficking (Cvrčková et al., 2024). The modulation of vacuole organization by bi-functional proteins linking membranes to the cytoskeleton was previously documented, e.g., for NET4, an actin-binding protein that is, at the same time, peripherally attached to the tonoplast (Kaiser et al., 2019; Hawkins et al., 2023). If this is also the case for AtFH1, the fraction of the protein residing at the tonoplast may fulfill some vacuole organization-related function.

To confirm this hypothesis, we investigated quantitative differences in tonoplast organization in the rhizodermis atrichoblasts of loss-of-function *fh1* mutants, presumed to produce no full-length AtFH1 protein (Oulehlová et al., 2019; Cifrová et al., 2020), in comparison to corresponding sister WT plants. We observed a reproducible trend toward an increased vacuole size and decreased vacuole shape complexity in the mutants, with the effect being statistically significant in small (meristematic and early transition zone) cells. This effect was also reproduced by treatment with SMIFH2, an inhibitor of formin-mediated actin nucleation, suggesting the participation of AtFH1-nucleated actin in central vacuole biogenesis. However, we cannot rule out the possibility that SMIFH2 may target additional proteins, as the inhibition of some families of myosins was reported in metazoan systems (Nishimura et al., 2021). However, there are currently no reports confirming off-target effects of SMIFH2 in plants, and Xu et al. (2024) recently ruled out the inhibition of myosin XIK by SMIFH2. The effects of actin disruption by LatB confirmed that central vacuole biogenesis depends on intact microfilament cytoskeleton in both WT plants and *fh1* mutants.

The documented vacuome rearrangements that appear to be affected by loss of AtFH1 or by the pharmacological inhibition of FH2 domain’s actin nucleation activity are relatively slow, typically taking place on the scale of hours. Our observations suggest that impairment of formin function advances, i.e., speeds up, these rearrangements. However, we also observed that *fh1* loss-of-function mutations, but not pharmacological formin inhibition, surprisingly impair rapid tonoplast motility that takes place on the seconds to minutes scale and is obviously connected to the cytoplasmic streaming. This suggests a possible separation of two tonoplast dynamic-related Class I formin functions: one modulating the slow ontogenetic movements, depending on FH2 domain-mediated, SMIFH2-sensitive actin nucleation; and the other involved in rapid tonoplast dynamics, and requiring physical presence (and possibly interactions) of the formin itself, regardless of its actin nucleation activity. However, without a detailed study of SMIFH2 effects on rapid tonoplast dynamics over time, involving also the investigation of the inhibitors’ pharmacokinetics, we cannot completely exclude the possibility of tonoplast dynamic alteration occurring after short-term inhibitor treatment but later eliminated either by inhibitor degradation or by some cellular adaptive mechanisms. The rapid, SMIFH2-insensitive tonoplast dynamics still requires an intact microfilament cytoskeleton, as documented by its inhibition by LatB, and the role of AtFH1 in this process may be mediated, for example, by its multiple but poorly characterized protein interactors (see Cvrčková et al., 2024) or by the (possibly subtle) alteration of actin cytoskeleton organization or membrane dynamics caused by formin function impairment. Indeed, changes in the expression level of another Class I formin, AtFH3, strongly affected F-actin cable organization and cytoplasmic streaming in pollen tubes (Ye et al., 2009). Interestingly, some Class I formins (although not AtFH1 so far) were found in association with clathrin-coated vesicles (Dahhan et al., 2022). Clathrin was demonstrated to contribute to ontogenetic vacuole rearrangements, although its possible involvement in rapid tonoplast movement has not been investigated (Osorio-Navarro et al., 2025) and would therefore be another interesting candidate whose role in formin-mediated vacuole dynamic changes may be worth further study.

The observed phenotypic effects of *fh1* mutations are, in general, subtle, probably due to functional overlap (redundancy) with other co-expressed formins, as already noted previously (e.g., Rosero et al., 2013, 2016; Cifrová et al., 2020). The apparent rootward shift (i.e., developmental advancement) of central vacuole formation in *fh1* mutants is in line with two previously reported phenotypic effects that could also be interpreted as developmental heterochrony. Loss of AtFH1 function notably enhances (i.e., advances) the development of pavement cell lobes in the cotyledon epidermis (Rosero et al., 2016) and increases the branching of epidermal trichomes (Cifrová et al., 2020). It would thus be tempting to speculate that AtFH1 may contribute to the regulation of developmental timing by a mechanism that is not yet known. However, a more parsimonious explanation of the observed phenotypes does not need to involve a specific regulatory function. Loss of AtFH1 was found to result in changes in cytoskeletal organization in space and time, namely, increased actin bundling and decreased actin dynamics in several cell types, including rhizodermis atrichoblasts, as well as increased microtubule motility (Rosero et al., 2013, 2016; Cvrčková and Oulehlová, 2017). The associated reduction in the amount of fine microfilaments may facilitate rearrangements of other cytoplasmic structures (Cvrčková et al., 2016), including the vacuole compartments.

Last but not least, local activities of tonoplast- and endosome-localized AtFH1, including actin nucleation as well as binding to other cytoplasmic components, may modulate the fate of AtFH1-carrying endomembrane compartments, possibly differentially on varying time scales, in agreement with our active cargo hypothesis (Cvrčková et al., 2024). Thus, our present study of the model Class I formin AtFH1 provides experimental evidence supporting the hypothesis that even the pathway toward degradation can be considered as an active part of a protein’s functional cycle.

## Data Availability

The datasets presented in this study can be found in the Zenodo repository, https://doi.org/10.5281/zenodo.16812971, and in the article/**Supplementary Material**.

## Supplementary material

Supplementary Table 1 List of constructs, building blocks and plasmids used in this study.

Supplementary Table 2 Primers used in this study.

Supplementary Table 3 Transgenic *A.thaliana* lines used in this study.

Supplementary Figure 1 Vacuolar lumen fluorescence of AtFH1-mScarlet-I in the meristematic and early transition zones.

Supplementary Figure 2 Effect of WM treatment on AtFH1 localization in an additional genetic background.

Supplementary Figure 3 Comparison of vacuole organization in WT and *fh1–4* seedling atrichoblasts.

Supplementary Figure 4 Effects of SMIFH2 treatment on vacuole organization in atrichoblasts of WT sister segregants of the *fh1–4* line.

Supplementary Figure 5 Comparison of tonoplast motility in WT and *fh1–4* atrichoblasts.

Supplementary Video 1 Tonoplast motility in the rhizodermis of a WT plant expressing the tonoplast marker VHP1:mGFP (*.avi).

Supplementary Video 2 Tonoplast motility in the rhizodermis of a *fh1:CRISPR* plant expressing the tonoplast marker VHP1:mGFP (*.avi).
