# Supplementary material for: The fate and function of the Arabidopsis Class I formin AtFH1 during central vacuole biogenesis in the rhizodermis
Source: Front Plant Sci. 2025 Oct 21;16:1685260. doi: 10.3389/fpls.2025.1685260 (PMC12583038; doi:10.3389/fpls.2025.1685260)
Supplement: Supplementary Table 1 — List of constructs, building blocks and plasmids used in this study. [file DataSheet1.pdf]

Supplementary Material

1 Supplementary Tables

Table S1: List of constructs, building blocks and plasmids used in this study.

Construct name	Author/Source	Insert	Note
**Prepared constructs**			
pLX::Pink::BastaR	This work	Pink::BastaR	Backbone with inserted plant selection cassette for further cloning
pLX::pFH1-FH1-mScarlet::BastaR	This work	pFH1:FH1:mScarlet:Ubq3Ter::BastaR	Final construct for plant transformation
pLX::pFH1-FH1-TagBFP::BastaR	This work	pFH1:FH1:BFP:Ubq3Ter::BastaR	Final construct for plant transformation
**Building blocks used for cloning**			
pUPD1-UBQ3Ter	Nelson Serre, Matyáš Fendrych	UBQ3Ter	Terminator
pUPD2::mScarlet B5	Shiv Mani Dubey, Matyáš Fendrych	mScarlet	mScarlet B5 (C-term fusion)
pUPD2::TagBFP B5	Tomáš Moravec	TaqBFP	TagBFP B5 (C-term fusion)
pLX delta	Tomáš Moravec	Empty vector	Low copy omega/delta alternative, derived from Pasin et al., 2017; Moravec et al., manuscript in preparation.
alpha2::35S:BastaR	Tomáš Moravec, Eliška Kobercová	35S:BastaR	Derived from Paz et al. 2006 by Tomáš Moravec
α13 pink	Tomáš Moravec	Pink casette	Moravec et al., manuscript in preparation
α11 sf	Dusek et al., 2020	stuffer fragment	
α12 sf	Dusek et al., 2020	stuffer fragment	
α14 sf	Dusek et al., 2020	stuffer fragment	
**Other**			
FH1:GFP	Oulehlová et al., 2019	pFH1-FH1-genomic	

Table S2: Primers used in this study.

Primer name	Sequence 5´- 3´	Purpose	Source
FH1-delta-F1-v6	TGCGCGGTCTCGGGAGCATTAATTATTAAGTGGACATGTGGC	pFH1:FH1-genomic cloning, part 1	This work
FH1-delta-R1-v4	TGCGCGGTCTCGGGGACCGAACAAATCTATAATCGGTT		
FH1-delta-F2-v4	TGCGCGGTCTCGTCCCCGTCACTGTCGTTAGCTTCTTT	pFH1:FH1-genomic cloning, part 2	
FH1-delta-R2-v4	TGCGCGGTCTCGGCCTCGAGATTGTGTCCGCTTTAGTA		
FH1-delta-F3-v4	TGCGCGGTCTCGAGGCCGCCTTCTCTTACACCGCCTTC	pFH1:FH1-genomic cloning, part 3	
FH1-delta-R3-v4	TGCGCGGTCTCGGAAGACCTAGTTTCCGGCATTTAATG		
FH1-delta-F4-v4	TGCGCGGTCTCGCTTCAAGTTGTATCAAGTCTCTGTTCTGA	pFH1:FH1-genomic cloning, part 4	
FH1-delta-R4-v4	TGCGCGGTCTCACGAACCAGAAACTAATGAGATTGAGTTATGTT		
pLX_seq_F	CAAAACCGGCTCAGTTCTGCG	Sequencing of pFH1-FH1-mScarlet	
pFH1_seq_F	ATCAAGTTATCAGACCGCAC		
pFH1_seq_F2	CTTAACATTCACAGGCCCAC		
FH1_seq_F1	CATCCTCCGATCTAGTCTTC		
FH1_p1-p2_seq_F	TTACTCTCCACGTGGCTCAC		
UBQ3Ter_R	GTACGGCCCATCTTCACCA		
Ubq3Ter_seq_F	GTTCACTACTACTCATTGACC		
F_AtFH1_RT5_2	ACTTACCAGTGACTTCGTCTC	Sequencing of pFH1-FH1-mScarlet	This study (designed by Eva Kollárová)
fh1-2 LP	TGTTTGTGTAGGCTGCTTGTG		Rosero et al, 2013
fh1-4RP	CCAAGCTTAACAGGCGAAT	Detection of *fh1-4* allele	Oulehlová et al., 2019
fh1-4LP	GAGTCAGGTGACTACTAAAGC		
Salk Lbb1.3	ATTTTGCCGATTTCGGAAC		http://signal.salk.edu/tdnaprimers.2.html
F_FH1_g4g7	CTATCTCCGCCGTTTCCTCC	Detection of *fh1:CRISPR* allele	Cifrová et al., 2020
R_FH1_g4g7seq1	AGATAACCGGCGGTTTTGGT		

Table S3: Transgenic *A.thaliana* lines used in this study.

Line	Back-ground	Source	Figures and videos	Note
fh1:CRISPR	Col-8	Cifrová et al., 2020		Parent line for crosses and transformations.
fh1-4	Col-0	Oulehlová et al., 2019		Parent line for crosses and transformations.
fh1-4/AtFH1-GFP	Col-0	Oulehlová et al., 2019	Fig. S2	
VHP1:GFP	Col	Segami et al., 2014		Parent line for crosses; donated by Falco Kruger and Melanie Krebs; not clear which Col derivative
fh1-4/VHP1:GFP	Col	This study (crossing)	Fig. S3, Fig.S5	
WT(*fh1-4*)/ VHP1:GFP	Col	This study (crossing)	Fig.8, Fig.S2, Fig.S4, Fig.S5	FH1 WT segregant from the *fh1-4* x VHP1:GFP cross
fh1:CRISPR/ VHP1:GFP	Col	This study (crossing)	Fig.4, Fig.6, Fig.7, Video S2	
WT(*fh1:CRISPR*)/ VHP1:GFP	Col	This study (crossing)	Fig.4, Fig.5, Fig.6, Fig.7, Video S1	FH1 WT segregant from the *fh1:CRISPR* x VHP1:GFP cross
fh1:CRISPR/ AtFH1-GFP	Col-8	Cvrčková et al., 2024	Fig.1A, Fig.2, Fig.3B	Independent transformants with varying signal intensity.
fh1:CRISPR/ AtFH1-mScarlet-I	Col-8	This study (floral dip)	Fig.1A, Fig.1B, Fig.3A, Fig.3B, Fig. S1	Three independent transformants, somewhat shorter root than parent line
fh1-4/ AtFH1-mScarlet-I	Col-0	This study (floral dip)	Fig.1A, Fig.1B, Fig.S2	Three independent transformants, phenotypically normal
fh1-4/VHP:GFP/ AtFH1-mScarlet-I	Col-0	This study (floral dip)	Fig.1A, Fig.1C	Phenotypically normal
fh1-4/VHP:GFP / AtFH1-TagBFP	Col-0	This study (floral dip)	Fig. 1A	T1 plant shown

2 Supplementary Figures

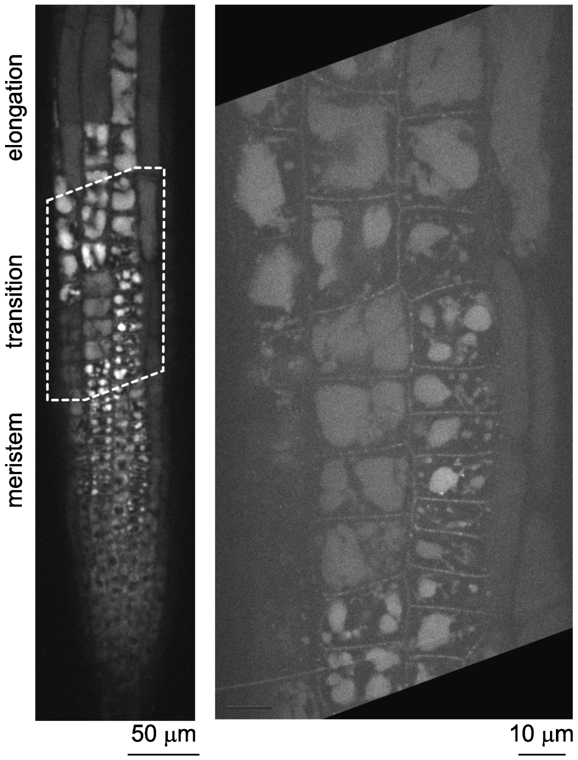

Figure S1. Vacuolar lumen fluorescence of AtFH1-mScarlet-I in the meristematic and early transition zones. Single low and high magnification confocal sections from a *fh1:CRISPR* background transgenic seedling root (a DMSO-treated control from a WM treatment experiment), showing approximate zonation. The two images shown were taken several minutes apart (lower magnification image first, higher magnification one taken immediately after objective change). Note the decrease in vacuolar fluorescence intensity in the small cells, indicating fast bleaching possibly combined with protein degradation. Fluorescence in small cells was near entirely lost within a few seconds of subsequent laser exposure. The dashed frame in the left panel marks the meristematic/early transition zone area shown at high magnification in the right one.

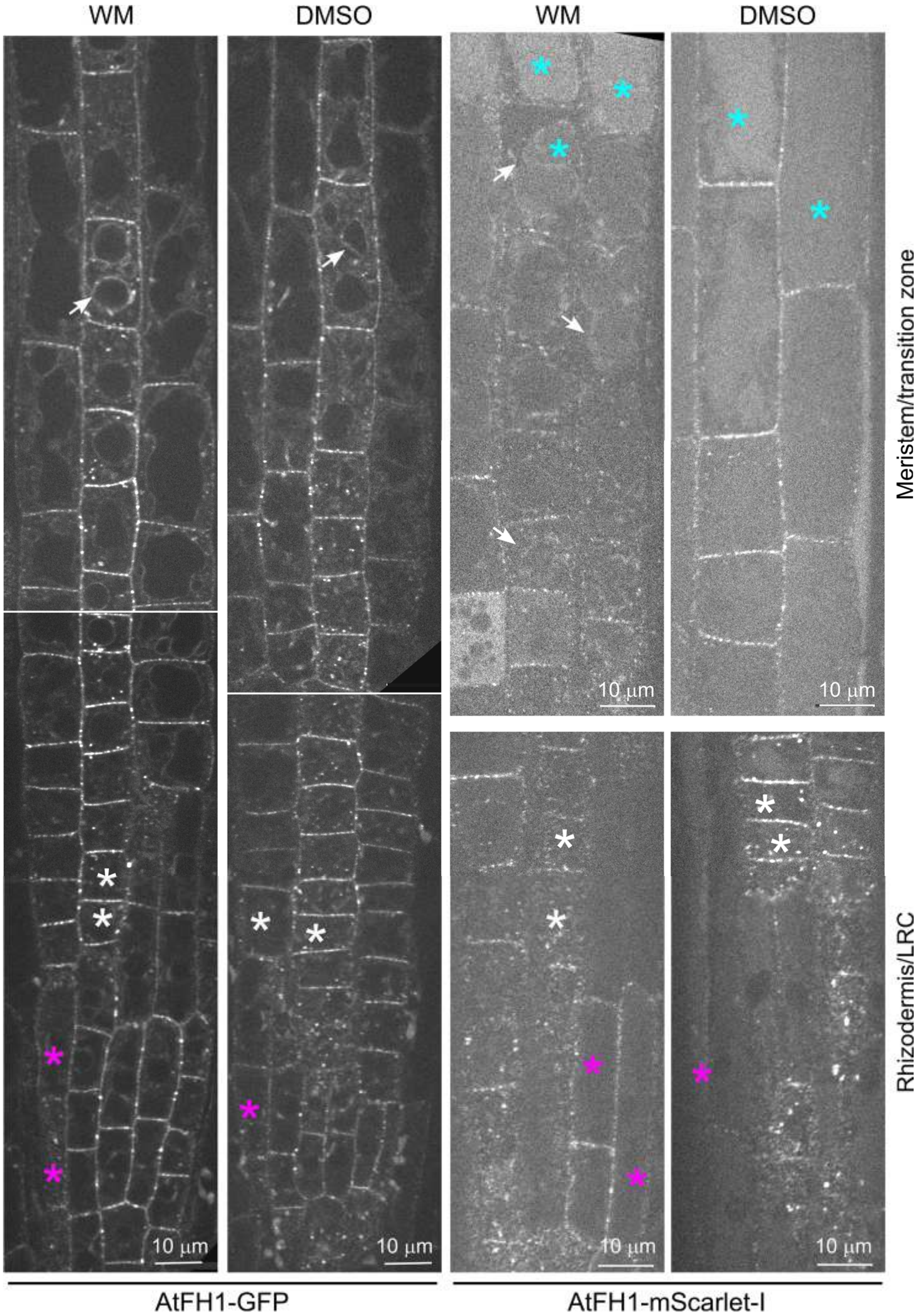

Figure S2. Effect of WM treatment on AtFH1 localization in an additional genetic background. Single confocal sections from 5 days old WM-treated and control (DMSO) treated *fh1-4* background transgenic seedlings are shown. Examples of vacuolar lumen fluorescence are marked by cyan asterisks, LRC cells by magenta asterisks, rhizodermis cells by white asterisks, tonoplast-localized AtFH1 by white arrows.

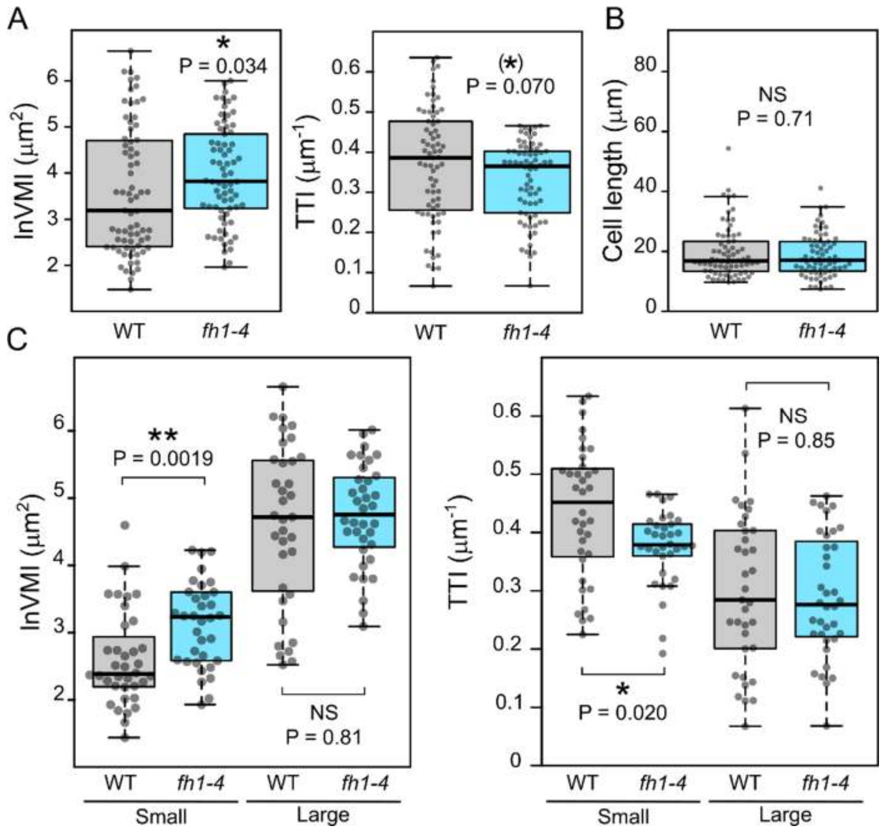

Figure S3. Comparison of vacuole organization in WT and *fh1-4* seedling atrichoblasts. (A) Quantitative characterization of tonoplast organization in atrichoblasts of WT and *fh1-4* plants expressing the tonoplast marker VHP1:mGFP, as determined by the VMI and TTI metrics. The evaluated roots grew either on the agar medium surface or embedded in the medium, in approximately equal proportion for both genotypes. (B) Comparison of cell length distribution of the WT and mutant atrichoblasts evaluated in (A), documenting comparable cell size in both samples. (C) Quantitative characterization of tonoplast organization in atrichoblasts as in (A), with cells split into two size categories - small (below median length) and large (above median length). Significance of between-genotype differences is documented by Mann-Whitney P-values, in (C) corrected for multiplicity using the Benjamini-Hochberg method.

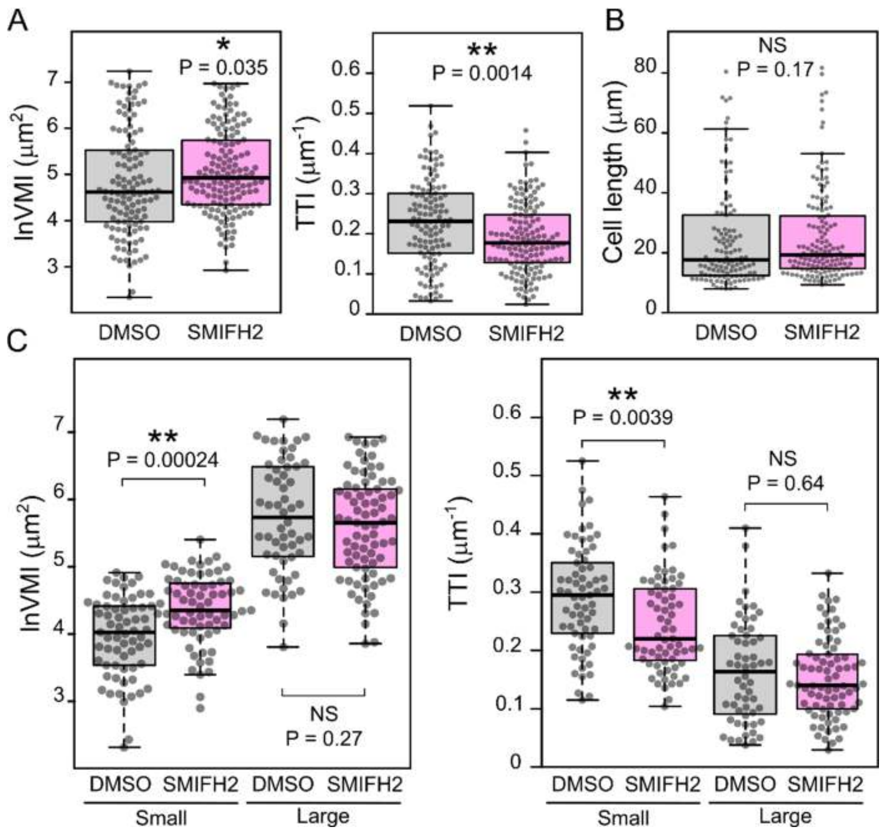

Figure S4. Effects of SMIFH2 treatment on vacuole organization in atrichoblasts of WT sister segregants of the *fh1-4* line. (A) Quantitative characterization of tonoplast organization in atrichoblasts of plants expressing the tonoplast marker VHP1:mGFP, grown on control (DMSO-containing) and SMIFH2-containing media, as determined by the VMI and TTI metrics. (B) Comparison of cell length distribution of the control and SMIFH2-treated plant atrichoblasts evaluated in (A), documenting comparable cell size in both samples. (C) Quantitative characterization of tonoplast organization in atrichoblasts as in (A), with cells split into two size categories - small (below median length) and large (above median length). Significance of between-genotype differences is documented by Mann-Whitney P-values, in (C) corrected for multiplicity using the Benjamini-Hochberg method.

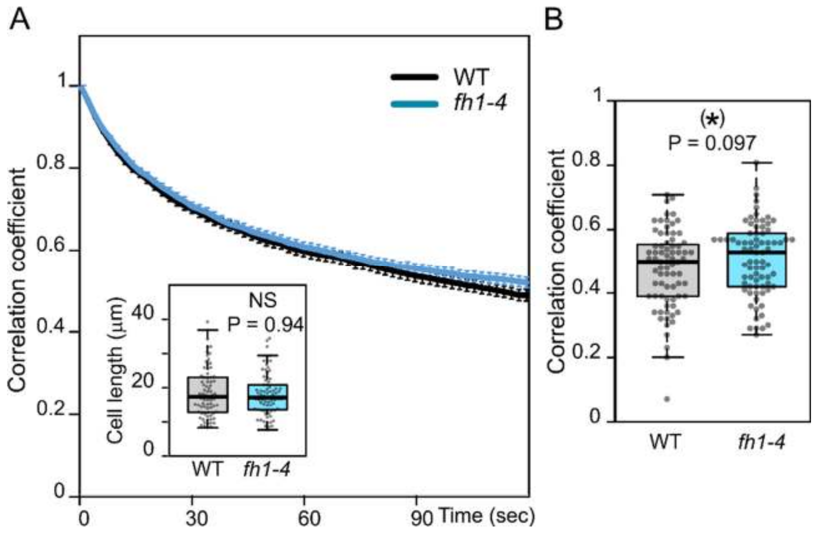

Figure S5. Comparison of tonoplast motility in WT and *fh1-4* atrichoblasts. (A) Average frame to 1st frame pixel intensity correlation coefficients (from at least 40 cells per genotype and time point), plotted against time. Error bars denote SEM. Inset: cell length distribution of the analysed cells, documenting comparable cell size in both samples. The evaluated roots grew either on the agar medium surface or embedded in the medium, in approximately equal proportion for both genotypes. (B) Distribution of average single cell pixel intensity correlation coefficients among two frames taken 120 seconds apart for each genotype, i.e., corresponding to the rightmost end of plots in (A), . Significance of between-genotype differences or lack thereof is documented by the Mann-Whitney P-value in (A) and by the t-test P value in (B).

3 Supplementary Movies

Movie S1. Tonoplast motility in the rhizodermis of a WT plant expressing the tonoplast marker VHP1:mGFP. Frame interval 2 seconds.

Movie S2. Tonoplast motility in the rhizodermis of a *fh1:CRISPR* plant expressing the tonoplast marker VHP1:mGFP. Frame interval 2 seconds.
